# Growing older with health and vitality: a nexus of physical activity, exercise and nutrition

**DOI:** 10.1007/s10522-016-9637-9

**Published:** 2016-02-15

**Authors:** Oliver C. Witard, Chris McGlory, D. Lee Hamilton, Stuart M. Phillips

**Affiliations:** Health and Exercise Science Research Group, University of Stirling, Stirling, FK9 4LA Scotland; Exercise and Metabolism Research Group, Department of Kinesiology, McMaster University, Hamilton, ON L8S 4K1 Canada

**Keywords:** Healthy ageing, Muscle mass, Protein intake

## Abstract

The preservation of skeletal muscle mass and strength with advancing age are, we propose, critical aspects of ageing with health and vitality. Physical inactivity and poor nutrition are known to accelerate the gradual age-related decline in muscle mass and strength—sarcopenia—however, both are subject to modification. The main purpose of this review is to present the latest, evidence-based recommendations for physical activity and exercise, as well as diet for older adults that would help in preserving muscle mass and strength. We take the position that future physical activity/exercise guidelines need to make specific reference to resistance exercise and highlight the benefits of higher-intensity aerobic exercise training, alongside advocating older adults perform aerobic-based physical activity and household tasks (*e.g.,* carrying groceries). In terms of dietary recommendations, greater emphasis should be placed on *optimal* rather than *minimum* protein intakes for older adults. Indeed, guidelines that endorse a daily protein intake of 1.2–1.5 g/kg BM/day, which are levels 50–90 % greater than the current protein Recommendation Dietary Allowance (0.8 g/kg BM/day), are likely to help preserve muscle mass and strength and are safe for healthy older adults. Being cognisant of factors (e.g., reduced appetite) that may preclude older adults from increasing their total daily protein intake, we echo the viewpoint of other active researchers in advocating that protein recommendations for older adults be based on a per meal approach in order to maximize muscle protein synthesis (MPS). On this basis, assuming three meals are consumed daily, a protein dose of 0.4–0.5 g/kg BM should be contained in each meal. We are beginning to understand ways in which to increase the utilization of ingested protein for the stimulation of MPS, namely by increasing the proportion of leucine contained in a given dose of protein, co-ingesting other nutrients (e.g., carbohydrate and fat or supplementation with n-3 polyunsaturated fatty acids) or being physically active prior to protein intake. Clearly, developing simple lifestyle interventions targeted at preserving muscle mass and strength with advancing age is crucial for facilitating longer, healthier lives into older age.

## Population ageing: a cause for celebration, concern or both?

Population ageing is widespread across Europe and worldwide. The percentage (median value across all European Union-27 countries) of Europeans aged >65 years increased from 12 % in 1985 to 17 % in 2010 (Office for National Statistics 2012). With the rate of avoidable mortality among people aged 60+ years old continuing to fall (Mathers et al. 2015), by 2035 ~25 % of Europe’s total population is projected to be >65 years old (Office for National Statistics. 2012). Fundamentally, this ageing demographic is testament to continued improvements in healthcare services. However, a disproportionate use of healthcare services by older people highlights the economic cost of population ageing, alongside the high prevalence of morbidity that can reduce quality of life with advancing age. As a consequence, there is a perception that society (in some European countries more than others) often views older people as a societal and economic burden. An opposing viewpoint is the socio-economic implications of human longevity are often misrepresented (Lloyd-Sherlock et al. 2012). The position of the present review is that advancing age does not necessarily have to be synonymous with poor health. Instead, older people remain capable of making significant social, economic and cultural contributions to society. Moving forward, ‘ageing well’ and a ‘healthy and independent life expectancy’ are two concepts that are being championed as priority public health messages by influential bodies such as the World Health Organization. As such, there are calls to prioritize ‘health span’, but exactly how this aim can be achieved remains an interesting topic of research. We view, as essential practices in ageing well, being physically active, a structured exercise programme and following good nutrition practice.

## Sarcopenia and the threat of physical disability

Sarcopenia is a late-life multifactorial syndrome with the potential to curtail quality of life and increase risk for physical disability. According to the consensus operational definition of the European Working Group on Sarcopenia in older people (EWGSOP) (Cruz-Jentoft et al. 2010), diagnosis of sarcopenia is characterized operationally by a low skeletal muscle mass, accompanied by low muscle strength and/or low physical performance. Whilst other definitions of sarcopenia have been proposed, all definitions have as essential elements a reduction in muscle mass, strength, and mobility. A gradual, but progressive, decline in skeletal muscle size and quality is an inevitable consequence of advancing age. The onset of muscle mass loss typically begins by the fourth decade of life (Janssen et al. 2000), having peaked between 20 and 30 years of age. Thereafter, the average rate of muscle mass loss is estimated at 8 % per decade (~0.5–1 %/year) until the age of 70 years (Mitchell et al. 2012), increasing to ~15 % per decade in octogenarians and beyond (Delmonico et al. 2009). Hence, most individuals 70–80 years old possess only 60–80 % of the muscle mass they had at ~30 years old, declining to <50 % in octogenarians.

Alarmingly, compared to muscle mass loss, the trajectory of strength loss is even more precipitous, with annual declines of 3–4 % reported in men and 2.5–3 % in women (Goodpaster et al. 2006). The clinical implications of sarcopenia include functional impairments (e.g. slow walking speed, poor balance) (Landi et al. 2012b, 2013) and, in severe cases, physical disabilities (e.g. difficulty performing activities of daily living, increased risk of falls) (Janssen et al. 2002), increased risk of cardiovascular and metabolic disease (e.g. type 2 diabetes and obesity) (Srikanthan et al. 2010) and all-cause mortality (Landi et al. 2012a). Conceptually, cutpoints of skeletal muscle mass index (appendicular muscle mass ÷ body mass) have been identified, below which the risk for physical disability increases in older adults (Janssen et al. 2004). This threshold assists clinicians in predicting the likelihood of functional impairments and physical disabilities across different ageing subpopulations. In the United States (no equivalent European data are available), it is estimated that 5 % of 65 year olds and 50 % of 80+ year olds suffer from sarcopenia (Morley 2012). Due to a lower starting muscle mass and energy intake, prevalence estimates are greater in women compared with men (Janssen et al. 2002). Taken together, this body of evidence highlights the potential detrimental impact of sarcopenia on functional independence and metabolic health and emphasises the necessity to try and slow the decline in skeletal muscle mass throughout life, but particularly in a person’s latter years.

**Fig. 1:**
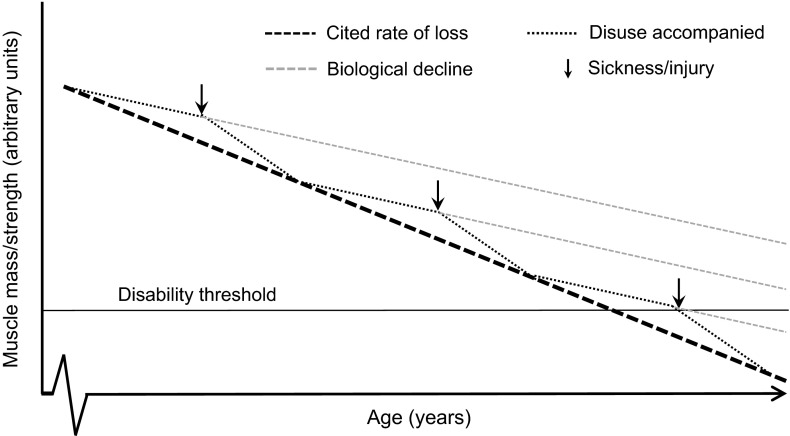
The decline in muscle mass and strength with advancing age. This figure has been designed to highlight the debilitating impact sporadic bouts of muscle disuse have on cited rates of muscle loss. The *solid black line* is the disability threshold; the *broken black line* is the mean cited rate of decline, the *broken grey line* is the biological decline and the *black dotted line* is the dis-use accompanied decline. The *arrow* refers to a period of disuse or reduced physical activity. Redrawn, with permission, from English and Paddon-Jones (2010)

One important, but often overlooked, consideration is that the cited rate of muscle mass and strength loss in older adults does not occur in a linear fashion. In fact, we and others (English and Paddon-Jones 2010) theorise that the natural decline in muscle mass and function with advancing age is punctuated by sporadic, but potentially inevitable bouts of muscle disuse (Fig. 1). These periods of disuse can result from a variety of situations, including convalescence, prolonged inclement weather (e.g., extreme heat or cold and snow), and hospitalization/bedrest due to injury or illness. Irrespective of causality, disuse independently results in skeletal muscle atrophy at any age (Paddon-Jones 2006; Wall et al. 2015); thus, in older individuals who experience protracted and/or frequent periods of disuse, normal age-related declines in muscle mass and function act synergistically with muscle disuse to accelerate the losses of muscle mass and strength (Fig. 1). Indeed, even two-weeks of reduced daily steps (<1000) has been shown to result in a decline of bone and fat-free mass, suppress postprandial rates of muscle protein synthesis (MPS), and reduce muscle function in older adults (Breen et al. 2013; Devries et al. 2015). Tackling the cause of such events remains a great challenge in both the scientific and built environment but, despite many years of work, we are still unaware of the distinguishing physiological mechanisms underpinning muscle disuse atrophy and sarcopenia. Whilst both sarcopenia and muscle disuse possess many mutually exclusive characteristics, there are some characteristics that are consistent observations in both conditions. Although we will review some common observations with ageing, sarcopenia, and muscle disuse, we acknowledge that a detailed discussion of the cellular and molecular mechanisms of muscle disuse atrophy and sarcopenia are not within the scope of the present article. Instead, we refer the interested reader to other informative papers (Cohen et al. 2015; Phillips et al. 2009).

## Aetiology of age-related muscle loss

The underlying causes of sarcopenia are multifactorial, interconnected and complex. Contributing processes include, but are not limited to: activity levels, nutrition, chronic inflammation, DNA damage, elevated oxidative stress, mitochondrial dysfunction, and changes in hormonal milieu (Morley 2012). Ultimately however, a net muscle protein balance (NBAL) favouring rates of muscle protein breakdown (MPB) over MPS underpins the age-associated decline in muscle mass. In this regard, studies have shown no clear difference in basal postabsorptive rates of MPS between young and older adults (Cuthbertson et al. 2005; Volpi 2001). Whilst the suppressive action of insulin on MPB has been reported to be diminished in older adults (Wilkes et al. 2009), a key mechanism proposed to underpin age-related muscle loss is *anabolic resistance*: defined here as a reduced ability of skeletal muscle to mount a *youthful* response of MPS to ingesting meal-like quantities of protein (Cuthbertson et al. 2005; Guillet et al. 2004) and/or other normally anabolic stimuli such as muscle loading (Durham et al. 2010; Fry et al. 2011; Kumar et al. 2009). Over time, anabolic resistance would, we (and others) propose, contribute to the gradual loss of muscle mass with advancing age via several mechanisms detailed below (Phillips et al. 2012).

The underlying cause of anabolic resistance can be explained at both cellular and molecular levels. The stimulation of MPS is fundamentally regulated by the availability of amino acids (Kimball and Jefferson 2002). Several metabolic factors, both at the cellular and molecular level, explain the age-related decline in amino acid availability and thus anabolic resistance. At the cellular level these factors include, but are not limited to, dysfunctional gut processes involving protein/amino acid digestion, absorption and transport kinetics, an increased retention of amino acids within splanchnic tissues (Boirie et al. 1997; Volpi et al. 1999) and impairments in microvascular perfusion (capillary recruitment and dilation). Hence, a relative hypoaminoacidemia (reduced amino acid availability) in older persons to ingesting a given quantity of protein has been proposed to result in a diminished capacity for stimulation of MPS.

At the molecular level, proposed mechanisms can be categorised into impaired translational efficiency (Cuthbertson et al. 2005; Guillet et al. 2004) and translational capacity (Kirby et al. 2015). With regards to both translational efficiency and translational capacity, some studies show that activation of the mechanistic/mammalian target of rapamycin (mTOR)/p70s6 kinase 1 (p70s6K1) signalling pathway—an integral signalling axis that regulates MPS—may be impaired in older adults in response to both protein feeding and exercise (Cuthbertson et al. 2005; Guillet et al. 2004). However, other studies in fact show an enhanced activation of these pathways in older muscle in response to resistance exercise or feeding (Farnfield et al. 2012; Mayhew et al. 2009). Furthermore, a systematic analysis of the basal activation of these pathways in young and old muscle demonstrates that they are higher in the fasted state in older individuals (Markofski et al. 2015). Thus, there appears to be discordance between the molecular pathways that control MPS and the actual rates/capacity for stimulation of MPS in older muscle. One interpretation could be that a molecular *block* elsewhere exists in a place other than between mTOR and p70s6K1 in the pathway(s) from sensing nutrition/mechanical strain and the molecular induction of a protein synthetic response. Ultimately, it appears that older muscle does have a reduced ability to transduce nutritional and loading signals and thus stimulate MPS, however the precise location of this reduced signal transduction is not known.

 With regards to the nutritional stimulation of MPS, amino acid availability is modulated by several dietary factors, including the essential amino acid (EAA) content of the protein source, amount of protein ingested (as a single dose), timing, pattern and coingestion of other nutrients. In addition, physical activity/exercise enhances the ability of skeletal muscle to respond to amino acid provision (Pennings et al. 2011a, b; Timmerman et al. 2012). In this regard, the most likely contributing mechanism is an exercise-induced increase in blood flow to the muscle (Biolo et al. 1997) that increases the delivery of amino acids to the muscle, thus increasing the provision of substrate for MPS (Biolo et al. 1997). Conceptually, interventions that enhance postprandial amino acid utilization in the ageing population would be beneficial. Whilst some pharmaceutical anabolic agents, such as testosterone, growth hormone and dehydroepiandosterone therapy have been shown to counteract muscle loss, their utility is limited due to a poor side-effect profile (Osterberg et al. 2014; Rolf and Nieschlag 1998). Thus, identifying simple (and sustainable) non-pharmacological lifestyle interventions targeted at preserving muscle mass and function are crucial for facilitating longer, healthier lives into old age. Physical activity and nutrition offer the most promising candidates and, in the context of this review, are considered to be at the nexus of getting older with health and vitality.

## Refining physical activity and exercise guidelines

Physical activity is associated with a panoply of health benefits including, but not limited to, improved cardiovascular fitness and strength, as well as a reduced risk for multiple chronic diseases. Current guidelines for physical activity in the United Kingdom recommend that adults 65+ years old should aim to perform 30 min of physical activity, in bouts of 10 min or more, at least 5 days per week (National Institute for Health and Clinical Excellence 2009). These guidelines include brisk walking, ballroom dancing, climbing stairs and running as examples of physical activity. Additional suggestions, in terms of physical activity modality, have been made for improving muscle strength of older adults. For instance, carrying or moving heavy loads such as groceries, as well as weight-bearing activities that involve stepping and jumping (i.e., dancing). As such, current physical activity guidelines for older adults take a broad approach encompassing a diversity of activities, ranging from everyday tasks to organised recreational activities. These guidelines are, in our view, somewhat ambiguous and non-specific for older adults. A conspicuous omission from these guidelines is the specific recommendation of structured resistance exercise for promoting the retention of strength and balance and an associated improvement in the health and vitality of older adults (Ferreira et al. 2012).

Resistance exercise has been widely used to enhance muscle mass and strength in a variety of populations (Peterson et al. 2010), including adults older than 75 years of age (i.e., those at greatest risk of anabolic resistance) who also display a marked ability to increase muscle mass and strength in response to resistance exercise training (Churchward-Venne et al. 2015). For example, in one study, 24 weeks of resistance exercise training in healthy elderly men and women significantly increased leg muscle mass by ~10 %, type 2 muscle fibre cross sectional area by ~25 % and one repetition maximum strength by ~40 %, and as well as reducing sit-to-stand time by ~18 % (Leenders et al. 2013). Other researchers investigating the impact of structured resistance exercise on skeletal muscle strength and morphology have observed similar results (Churchward-Venne et al. 2015). It is important to note that varying degrees of hypertrophy are often apparent between individuals, with those exhibiting the least gains commonly referred to as ‘non-responders’ (Davidsen et al. 2011). However, in a recent retrospective analysis from three separate resistance exercise-training studies, Churchward-Venne and colleagues (Churchward-Venne et al. 2015) demonstrated that there are in fact, no true *non*-*responders* to resistance training. What these authors show is that, whilst some older men and women failed to show significant improvement in an end-point measure such as muscle mass in response to a given resistance exercise protocol, they did show improvements in other clinically relevant parameters such as strength. Moreover, these improvements were positively associated with the duration of training (i.e., the longer the training the greater the improvements). Thus, refraining from resistance exercise in the belief that someone may not sufficiently ‘respond’ is currently an unsubstantiated assertion. Based on the available evidence, we therefore propose that resistance exercise should become a critical component of any future refined directives aimed at improving the functional independence of older adults.

Whilst the beneficial impact of resistance exercise on skeletal muscle mass and strength has received significant scientific attention, less emphasis has been placed on the role of aerobic exercise for preserving muscle mass of older adults. Since walking is the preferred mode of physical activity for older adults (Booth et al. 1997), as reflected by higher participation rates compared with resistance exercise (Manson et al. 2002), aerobic-based physical activity may be considered an important, albeit underappreciated, intervention targeted at overcoming age-related muscle mass and strength loss. Traditionally, aerobic exercise has been associated with improved oxidative capacity (Short et al. 2003), glycemic control (Lukacs and Barkai 2015) and cardiovascular fitness (Gibala et al. 2012). However, recent data also highlight the anabolic potential of aerobic-based exercise. For instance, one study reported a significant increase in quadriceps muscle volume and muscle power in older individuals who cycled for 20–45 min, at 60–80 % heart rate reserve, 3–4 times per week for 12 weeks (Harber et al. 2012). Similarly, older women who performed 12 weeks of progressive cycling showed increased quadriceps size (~12 %) and aerobic capacity (~30 %) (Harber et al. 2009). It is important to acknowledge that other studies have failed to detect an impact of aerobic exercise training on muscle mass in older adults (Short et al. 2004; Sipila and Suominen 1995). Such discrepant findings may be related to between-study differences in participant characteristics, such as training history and/or age, as well as exercise modality and intensity. Support for the latter contention arises from studies demonstrating that high intensity interval training (HIT) has the capacity to stimulate increases in MPS comparable to those of resistance-based exercise (Bell et al. 2015; Di Donato et al. 2014). Whereas continuous (low-intensity) aerobic-based exercise is characterised by exercise performed at a constant workload, HIT consists of brief, intermittent bursts of vigorous activity interspersed by periods of reduced activity or rest (Gibala et al. 2014). These bursts of activity are thought to be a key factor in stimulating MPS. A common misconception of HIT relates to health and safety, with the perception that HIT may render individuals, specifically those with cardiac issues, more susceptible to adverse cardiac events. However, closer inspection of available literature reveals that older individuals are at no greater risk when performing HIT compared to continuous training (Rognmo et al. 2012).

The physiological mechanisms underpinning the hypertrophic impact of aerobic exercise on skeletal muscle remain to be elucidated, but are thought to be distinct from those regulating resistance exercise-induced increases in muscle mass (Philp et al. 2015). Some evidence suggests that aerobic exercise improves nutrient delivery to skeletal muscle by resulting in an enhanced feeding-induced vasodilation and thus amino acid delivery. Indeed, Timmerman and colleagues (Timmerman et al. 2012) demonstrated that performing a bout of aerobic exercise (45 min on a treadmill at 60–70 % heart rate reserve) enhanced the rates of MPS in response to essential amino acid and sucrose ingestion 18 h later. A key mechanistic finding of this study was that the prior bout of aerobic exercise increased microvascular perfusion and amino acid delivery to the muscle. Given that anabolic resistance may be, in part, due to reduced amino acid delivery to the muscle (Boirie et al. 1997; Volpi et al. 1999), aerobic exercise could be a viable means to improve the anabolic impact of protein feeding. Thus, aerobic exercise not only appears to be effective at improving aerobic capacity, but also other clinically relevant health factors such as muscle mass, and in some instances, muscle strength. However, in order for adaptations to resistance or aerobic-based exercise to be sustained in the long-term, the adequate provision of nutrition, specifically dietary protein intake, is essential. In this regard, we review here the latest, evidence-based, nutritional recommendations for supporting musculoskeletal health with advancing age.

## Optimizing the quantity of protein recommended on a per meal basis

Several recent insightful reviews propose the most pragmatic approach to devising protein recommendations for older adults is to express protein intakes on a meal-by-meal basis, rather than the more traditional daily basis (Moore 2014; Paddon-Jones and Rasmussen 2009; Paddon-Jones et al. 2015). This argument is primarily driven by results from a series of acute metabolic studies, in both young and older adults, that characterised the relationship between ingesting a single dose of protein and the postprandial stimulation of MPS (Cuthbertson et al. 2005; Moore et al. 2009; Pennings et al. 2012; Robinson et al. 2013; Symons et al. 2009; Witard et al. 2014; Yang et al. 2012a, b). As such, the optimal meal protein intake represents the dose of protein that acutely stimulates a maximal postprandial response of MPS. These studies examined a range of protein sources, including intact proteins (whey, soy) and complete protein-rich foods (e.g. beef) at rest and during exercise recovery in either young (Cuthbertson et al. 2005; Moore et al. 2009; Witard et al. 2014) or older (Cuthbertson et al. 2005; Pennings et al. 2012; Robinson et al. 2013; Yang et al. 2012a, b) adults. In young adults (of ~75–80 kg body mass), the postprandial response of MPS reached a plateau with 20 g of ingested protein (Moore et al. 2009; Witard et al. 2014). Forty grams of protein stimulated non-anabolic processes such as increased rates of amino acid oxidation and urea production, but failed to potentiate an increased stimulation of MPS. By comparison, in middle- to older-aged adults, the protein dose-MPS response curve was shifted to the right and exhibited a lower gain per protein dose relative to young adults. The postprandial response of MPS continued to increase with ingestion of 35–40 g compared with 20 g of protein (Pennings et al. 2011b; Yang et al. 2012a). A growing consensus is this rightward shift is not mediated by biological age *per se*, but also is influenced by increased prevalence of muscle disuse due to reduced physical activity levels associated with advanced age. Nonetheless, whereas these collective data are unable to identify the optimal meal protein intake (expressed on an absolute basis) for maximal stimulation of MPS in older adults, it is clear that the optimal meal protein intake for older adults exceeds that of young adults. Interestingly, whereas a sex-specific difference in the response of MPS to exercise and protein ingestion is not seen in young adults (Dreyer et al. 2010; Scalzo et al. 2014; Smith et al. 2009), a sex-based difference in the postprandial response of MPS has been reported in older adults (Smith et al. 2008). Thus, although not directly evaluated, these data suggest that the optimal single bolus dose of protein in older adults may differ between men and women. Future studies are warranted to test this thesis and to fully establish the optimum meal protein intake for older adults.

Whereas the postprandial response of MPS to a single protein feed has received much attention, the aggregate daytime stimulation of MPS in response to multiple protein feeds (mimicking a typical daily meal feeding cycle) has only recently been investigated in young (Areta et al. 2013; Mamerow et al. 2014) and older (Kim et al. 2014) adults. Theoretically, the daytime stimulation of MPS depends, not only on stimulating a maximal response of MPS to each meal, but also on the spacing and frequency of daily meals. A recent study in young adults (Mamerow et al. 2014) demonstrated a greater 24 h response of MPS to a balanced meal pattern that distributed 90 g of protein evenly between three meals (3 × 30 g), spaced 3.5–4 h apart versus a conventional (Tieland et al. 2012; Valenzuela et al. 2013) skewed meal pattern that biased daily protein intake (~63/90 g) towards the evening meal. Thus, despite an equal total daily protein intake (90 g), the aggregate daytime stimulation of MPS was greater with a balanced meal pattern that spread daily protein intake evenly between meals. A theoretical explanation for this differential aggregate daytime response of MPS is a more favourable cyclical fluctuation pattern of aminoacidemia, in particular leucinemia, with a balanced pattern of protein ingestion (Mamerow et al. 2014). In light of this fluctuating pattern of amino acid availability, a threshold leucine availability was surpassed with each meal, switching on the muscle anabolic signalling proteins that stimulate MPS. Thus, there is preliminary evidence that distributing total daily protein intake evenly between meals offers an effective strategy to potentiate the aggregate daytime stimulation of MPS in young adults.

The impact of protein meal pattern on muscle mass regulation in older adults is not well established. Previous studies have collected measurements of (acute) muscle protein metabolism (Kim et al. 2014), whole body protein metabolism (Arnal et al. 2000; Kim et al. 2014) and/or (chronic) changes in muscle mass (Bouillanne et al. 2013) and reported equivocal findings. For example, an observational study (Bollwein et al. 2013) compared the habitual protein distribution pattern between frail and non-frail older adults of similar age and reported that frail older adults (i.e., those that suffered the most muscle loss) habitually consumed a large proportion of daily protein intake in a single meal, whereas non-frail adults (i.e., those suffering the least muscle loss) balanced their daily protein intake more evenly between meals. In contrast, an intervention-based study (Bouillanne et al. 2013) in institutionalised, frail older adults reported a greater increase in muscle mass when total daily protein intake was consumed primarily as one discrete lunchtime meal rather than a more balanced spread of daily protein intake between breakfast, lunch, afternoon snack and evening meals. These conflicting findings (Bouillanne et al. 2013) to results in young adults (Areta et al. 2013; Mamerow et al. 2014) may be reconciled by the quantity of protein contained in each meal of the balanced pattern (range: 12–21 g) being insufficient for the maximal acute stimulation of MPS in the older adults studies by Bouillanne et al. (2013). As we (Moore et al. 2014) and others (Pennings et al. 2011b; Yang et al. 2012a) have previously established the maximal per meal protein dose in older subjects is well beyond 21 g per meal.

A recent study (Kim et al. 2014) demonstrated that a balanced protein meal pattern, whereby each meal likely contained a sufficient quantity of protein (~37 g) for the maximal acute stimulation of MPS, conferred no advantage regarding the daytime stimulation of MPS in healthy older adults. However, this study (Kim et al. 2014) was likely underpowered given that the sample size of the unbalanced group was only four participants. In theory, older adults that follow a meal pattern spacing each optimal protein dose sufficiently far apart to allow for a repeated stimulation of MPS will delay the onset, or slow the progression, of muscle mass loss with advanced age (Paddon-Jones and Rasmussen 2009). On a practical level, modifications of daily protein distribution should target the breakfast meal that is often the meal with lowest protein content. However, to date no study has comprehensively investigated the influence of protein meal pattern, combined with resistance or aerobic-based physical activity, on the aggregate daytime response of MPS to multiple meals in older adults. Exciting recent developments in tracer methodology, namely the oral deuterium oxide (^2^H_2_O) method, allows for the measurement of integrated rates of MPS and MPB over prolonged periods (days to weeks) (Bell et al. 2015; MacDonald et al. 2013; Wilkinson et al. 2014) and thus is ideally suited to evaluating the impact of protein meal pattern on muscle protein metabolism over extended time periods.

Whilst data from acute metabolic dose-response studies provide the basis for informing recommendations regarding the optimal quantity of protein to consume in each meal or combination of meals, the practical application of these data has been questioned (Drummond 2015; Moore et al. 2014). Essentially, these studies plotted the postprandial response of MPS against set quantities (i.e., not adjusted for BM or lean body mass) of high-quality protein, ranging from 0 to 40 g. As discussed below, protein guidelines (*minimum* protein requirement and *optimal* protein recommendations) are typically expressed relative to total body mass. Thus, it may be argued that establishing an optimal meal protein intake on a relative basis (g/kg BM/meal) provides a more informative approach for older adults. Accordingly, a retrospective analysis of serial dose-response studies revealed the optimal relative dose of protein in a single serving for the maximal resting postprandial stimulation of myofibrillar-MPS to be 0.40 g/kg BM in older adults; ~68 % greater than calculated in young adults (0.24 g/kg body mass) (Moore et al. 2014). Thus, assuming three meals are consumed each day, a relative protein dose of 0.4–0.5 g/kg BM/meal is consistent with recent expert opinions concerning the optimal daily protein intake (1.2–1.5 g/kg BM/day) for healthy older adults (Bauer et al. 2013; Deutz et al. 2014), as discussed below.

## Optimizing the quality and utilization of protein intake on a per meal basis

In practice, the recommendation to consume larger (e.g. exceeding a meal-like portion) and frequent (3–4× day) protein feeds (≅30 g protein/meal for a 70 kg person at 0.4–0.5 g/kg BM/meal) may be challenging, or even counter-productive, for some older adults. Appetite is often suppressed with advancing age (Westenhoefer 2005). Several other factors, including physical and mental disabilities that limit shopping, food preparation and food insecurity due to financial and social limitations (Deutz et al. 2014) may also make it difficult for older adults to consume sufficient protein. To compound this issue, protein-rich foods exhibit greater satiety value than carbohydrate- or fat-rich food sources (Rolls et al. 1988). Hence, optimizing the *quality* of protein ingested in each meal also is considered to be of significant clinical value for offsetting age-related muscle loss.

In the context of muscle mass regulation, high quality protein refers to the efficient utilisation of ingested protein for maximising the postprandial stimulation of MPS (Millward et al. 2008). Protein quality is defined by the biological and physical properties of a protein source. Biological quality is dictated by two inter-related factors, namely the amino acid content and digestibility (and subsequent absorption kinetics) of the protein source that collectively determine amino acid bioavailability. As described previously, in terms of amino acid profile, the leucine content of a protein source is of particular importance for stimulating a postprandial response of MPS. Leucine not only provides substrate for the synthesis of new muscle protein, but also serves as a key anabolic signal for skeletal muscle by activating enzymes within the mTOR signalling pathway (Anthony et al. 2001). Indeed, the *leucine threshold* hypothesis (Rieu et al. 2006) has been proposed to explain the observation that young muscle appears relatively sensitive to the anabolic action of small (~1 g) quantities of ingested leucine, whereas older muscle requires ≥2 g of leucine (typically contained in ~20 g of high-quality protein) to increase MPS above resting rates (Phillips 2015). The physical property of protein sources refers to the food matrix (solid, semi-solid or liquid form). Food matrix also impacts amino acid digestibility (Conley et al. 2011). Thus, from a protein quality standpoint, it is commonly accepted that a rapidly digested and leucine-rich protein source is best positioned to activate mTOR and stimulate a postprandial response of MPS.

As already mentioned, leucine acts both as a trigger for initiating MPS, and as substrate for the synthesis of new muscle protein. Therefore, the efficient utilization of ingested amino acids for the postprandial stimulation of MPS depends, in part, on the leucine content of the protein source. Three lines of evidence suggest that enhancing the proportion of leucine contained in a protein source confers an anabolic advantage in older adults. First, two studies in older adults matched the dose of ingested EAA between conditions, but manipulated the leucine composition of the ingested EAA source (Dickinson et al. 2014; Katsanos et al. 2006). Results showed that ingestion of a leucine-enriched EAA feed amplified the resting postprandial stimulation of MPS (Katsanos et al. 2006) and prolonged the post-exercise (Dickinson et al. 2014) stimulation of MPS in response to ingesting a suboptimal dose (6.7–10 g) of EAA. Second, a recent study in older adults extended these findings by reporting a similar resting postprandial and post-exercise response of myofibrillar-MPS to ingestion of a leucine-enriched low dose of EAA (containing 1.2 g leucine; 3 g EAA) compared with feeding a 20 g bolus of whey protein (containing 2 g leucine; 9.6 g EAA) (Bukhari et al. 2015). These data corroborate, in part, earlier findings in young adults whereby ingestion of a leucine-enriched suboptimal (6.25 g) dose of whey protein stimulated a similar resting postprandial response of MPS compared with an optimal (25 g) dose of whey protein (Churchward-Venne et al. 2012). Thus, in terms of optimizing the utilisation of ingested amino acids for the postprandial stimulation of MPS, increasing the proportion of leucine contained in a suboptimal dose of protein offers a practical, low calorie and less satiating alternative to ingesting an appreciably larger amount of protein for some older adults. Future studies should provide a similar comparison between a leucine-enriched suboptimal protein dose (i.e., 20 g of whey protein) and an optimal protein dose (~40 g of whey protein) in older adults at rest and during exercise recovery. Finally, several studies in older adults have manipulated the leucine content of an amino acid source by providing additional leucine (Casperson et al. 2012; Koopman et al. 2008; Verhoeven et al. 2009; Wall et al. 2013). The addition of leucine (+2.5 g) to a meal-like feed of casein protein (20 g) increased the utilisation of ingested amino acids (Wall et al. 2013) for the postprandial stimulation of MPS (Rieu et al. 2006; Wall et al. 2013). In addition, 2 weeks of leucine supplementation increased the resting postabsorptive and postprandial response of MPS to a suboptimal dose of EAA plus carbohydrate (Casperson et al. 2012). However, the addition of leucine to a protein source does not necessarily enhance the muscle protein anabolic response. For instance, coingesting leucine with a ‘leucine-rich’ whey protein plus carbohydrate mixture failed to potentiate the response of MPS during exercise recovery (Koopman et al. 2005) and no chronic change in muscle mass was observed after 12 weeks of supplementing a moderate protein diet (~1.0 g/kg BM/day, ~6.3 g leucine per day) with additional leucine at each main meal (Verhoeven et al. 2009). Taken together, these data suggest the efficacy of supplemental leucine for modulating muscle mass in older adults depends on the leucine content of the original protein source or diet. Future work is warranted to investigate the influence of adding leucine to other isolated types of lower leucine-containing proteins, such as soy, rice of pea protein, on resting postprandial and post-exercise rates of MPS in older adults.

## Other nutrients may increase the utilisation of ingested protein

Since protein is typically coingested with carbohydrate and fat, it is important to understand the impact of macronutrient coingestion on the utilisation of ingested protein. The independent ingestion of carbohydrate and fat exerts negligible impact on MPS following exercise, at least in young adults, likely due to an insufficient availability of EAA (Borsheim et al. 2004; Miller et al. 2003). Moreover, when meal-like (~20 g) doses of protein are ingested, coingestion of moderate (40 g) or larger (60 g) doses of carbohydrate with protein does not increase the resting postprandial response of MPS in older adults (Gorissen et al. 2014; Hamer et al. 2013). The absence of an additive effect is evident despite a markedly greater increase in blood insulin concentrations with carbohydrate coingestion (Gorissen et al. 2014; Hamer et al. 2013). However, this observation may be limited to the response to ingestion of a moderate/large protein dose. A previous study in young adults suggests MPS may be enhanced when carbohydrates were ingested concurrently with a small dose of amino acids (containing ~6 g EAA) (Miller et al. 2003). Moreover, ingestion of whole milk has been shown to stimulate a superior response of NBAL compared with skimmed milk (both containing ~4 g EAA) in young adults (Elliot et al. 2006). Given that the only difference between conditions in this study (Elliot et al. 2006) was the fat content, these data suggest the combined ingestion of fat and carbohydrate may enhance the utilisation of a suboptimal amount of EAA for MPS or even suppress MPB. More recent results, also in young adults, are consistent with the notion that adding carbohydrate and fat to smaller amounts of EAA (in the form of an intact protein) may enhance the response of MPS (Churchward-Venne et al. 2012, 2014). Indeed, a leucine-enriched suboptimal dose of whey protein stimulated an inferior post-exercise response of MPS compared with an optimal dose of whey protein when consumed independent of other macronutrients (Churchward-Venne et al. 2012). However, coingesting carbohydrate and fat with a leucine-enriched suboptimal protein dose stimulated a similar post-exercise response of MPS compared with an optimal protein dose (Churchward-Venne et al. 2014). Taken together, these data (Churchward-Venne et al. 2012, 2014) suggest that carbohydrate and fat intake may impact the utilisation of ingested EAA for MPS following exercise, particularly if the amount of EAA is suboptimal. Clearly, more work is now needed, especially in older adults, regarding the impact of macronutrient co-ingestion on muscle anabolism both at rest and following exercise.

The efficacy of other nutritional interventions, such as supplementation with fish oil-derived n-3 polyunsaturated fatty acids (n-3PUFAs), for increasing the utilisation of ingested protein has been studied (Smith et al. 2011b, 2015). Indeed, 8 weeks of supplementation with a daily dose of 1.86 g of eicosapentaenoic acid (EPA) and 1.50 g of docosahexaenoic acid (DHA) was shown to potentiate rates of MPS in response to simulated feeding (a hyperaminoacidemic-hyperinsulinaemic clamp) in young, middle-aged and older adults (Smith et al. 2011a, b). The authors also identified this potentiated response of MPS with n-3 PUFAs was associated with enhanced mTORC-p70S6K1 phosphorylation, thus providing one potential mechanism of action. There also have been other reports corroborating the anabolic influence of n-3 PUFA consumption on skeletal muscle. Indeed, one such study supplemented older adults with 0.40 g/d of EPA and 0.3 g/d DHA during 90 days of resistance exercise training and reported greater strength gains in older women compared with a placebo (Rodacki et al. 2012). Moreover, expanding upon their initial work, Smith and colleagues recently demonstrated that daily ingestion of n-3 PUFAs (1.86 EPA and 1.50 DHA) increased handgrip strength and thigh volume in older men even in the absence of structured exercise training (Smith et al. 2015). The clinical implications of these data are particularly significant given that handgrip strength (Leong et al. 2015), and strength in general (Metter et al. 2002), are known predictors of all-cause mortality. The mechanism by which n-3 PUFA consumption confers this positive influence is proposed to be attributed to increases in the n-3 PUFA composition of muscle. In this regard, we have shown that fish oil supplementation (3.50 g g/d EPA and 0.9 g g/d DHA) results in a detectable increase in skeletal muscle n-3 PUFA composition within 2 wks in young men, and this increase is related to positive changes in the content of anabolic signaling molecules (mTOR, FAK) (McGlory et al. 2014). Thus, taken together, it appears that n-3 PUFA supplementation expands the n-3 PUFA composition of muscle that serves to prime muscle to respond to anabolic stimulation in the form of feeding and potentially resistance exercise.

As a result of these (Rodacki et al. 2012; Smith et al. 2015) and other investigations, n-3 PUFA supplementation is now receiving significant attention as a potential non-pharmacological therapy to combat skeletal muscle wasting and promote physical independence in a variety of settings. However, in our view, the aforementioned studies should be interpreted with a degree of caution. For instance, it is important to note that in the studies by Smith and colleagues (Smith et al. 2011a, b), the provision of amino acids was via an intravenous infusion, which is not indicative of habitual protein ingestion, (i.e., oral intake) and measurements of MPS were limited to a matter of hours and were not reflective of mixed meal consumption. Furthermore, in the work of Rodacki and colleagues (Rodacki et al. 2012), no placebo group was employed, the changes in n-3 blood composition were very small, and no direct measures of muscle mass and MPS were made. Even in our own study (McGlory et al. 2014), measured changes in the n-3 PUFA composition with n-3 PUFA ingestion were limited to 4 wks, using a single dose, made in the absence of changes of muscle function, and were conducted in a healthy young, male population. So, before population-wide recommendations can be made, future work is required, specifically relating to dose, to establish the efficacy of n-3 PUFA ingestion to enhance muscle mass and function in older adults. Nevertheless, these emergent data are promising and may provide a valuable means in conjunction with exercise to attenuate the age-related decline in muscle mass and function.

## Optimizing protein recommendations on a daily basis

Despite convincing arguments for communicating nutritional recommendations on a per meal basis, currently guidelines are expressed on a daily basis. In this regard, several recent reviews highlight the importance of distinguishing between the concepts of *minimum* and *optimum* daily protein intakes for older adults (Layman et al. 2015; Moore 2014; Rodriguez and Miller 2015). At present, the Recommended Dietary Allowance (RDA) for protein is set at 0.8 g/kg ideal body mass (BM)/day for healthy adults, regardless of age and sex. By characterising the minimum daily protein intake required to achieve nitrogen balance, this standard approach (i.e., the RDA) is intended to estimate the minimum daily protein requirement necessary to avoid negative nitrogen balance. However, the application of the protein RDA to informing the protein requirement of older adults has been criticised for several reasons other than the well documented methodological limitations associated with accurately quantifying all routes of nitrogen input and output (Bauer et al. 2013). First, the meta-analysis of long-term nitrogen balance studies used to determine the protein RDA primarily included studies in young adults (18/19 studies) (Rand et al. 2003). Hence, the RDA value for older adults has essentially been extrapolated from data generated in young adults. Second, the adequacy of the current protein RDA for maintaining whole body nitrogen equilibrium in older adults, in particular hospitalized patients (Gaillard et al. 2008), is not supported by some short-term nitrogen balance studies (Durham et al. 2010; Fry et al. 2011; Kumar et al. 2009). Indeed, a retrospective assessment of data in older adults estimated nitrogen equilibrium was achieved at a protein intake of 0.91 g/kg BM/day (Campbell et al. 1994), which is 15 % greater than the current RDA. In addition, studies using the indicator amino acid oxidation technique have reported a greater requirement for protein for older women (Rafii et al. 2015; Tang et al. 2014). Taken together, these lines of evidence suggest that even the protein RDA value of 0.8 g/kg BM/day underestimates the minimal protein requirement for older adults. In essence, we and others (Rodriguez and Miller 2015) propose that the terminology *RecommendedDietary Allowance* is misleading and likely contributes to the common misconception that the protein RDA is intended to guide a *recommended* protein intake that people are *allowed* to have, rather than *minimum* protein requirement for older adults. Accordingly, new recommendations from international study groups (Bauer et al. 2013; Deutz et al. 2014) propose the minimum daily protein intake for healthy older adults should be increased to 1.0–1.2 g/kg BM/day.

Evidence is accumulating that the optimum daily protein intake for older adults markedly exceeds the current RDA. International study groups (Bauer et al. 2013; Deutz et al. 2014) recommended 1.2–1.5 g protein/kg BM/day for adults older than 65 years. Three separate lines of evidence support this recommendation. First, two long-term intervention studies standardised the protein intake of healthy older adults at 0.8 g/kg BM/day (RDA) and reported a decline in mid-thigh muscle area after 14 weeks (Campbell et al. 2001, 2002). These data suggest the protein RDA is insufficient for healthy older adults to maintain skeletal muscle mass. Second, several larger-scale longitudinal studies reported that higher (>1.0 g/kg BM/day) daily protein intakes could offset the age-related decline in muscle mass (Gray-Donald et al. 2014; Houston et al. 2008; Meng et al. 2009; Stookey et al. 2005), strength (Bartali et al. 2012; Beasley et al. 2013; Scott et al. 2010) and physical performance (Gregorio et al. 2014; Vellas et al. 1997). Although causality cannot be established from observational studies, these data suggest older adults who consume a diet with protein intake higher than the RDA better preserve muscle size and functional ability. Finally, a recent intervention study demonstrated a greater daytime (22 h) MPS response with consumption of a protein intake equivalent to 2 × RDA (1.6 g/kg BM/day) compared with the RDA (0.8 g/kg BM/day) (Kim et al. 2014). Taken together, these data in older adults highlight the acute and chronic benefits of consuming a protein intake greater than the current RDA on MPS, muscle mass and muscle function. Future work is needed to refine the recommendation for an *optimum*, as opposed to minimum daily protein intake for musculoskeletal health in older adults.

Whilst the health benefits of recommending protein intakes above the RDA have received scientific support (Bauer et al. 2013; Deutz et al. 2014), concerns over potential negative health implications to kidney function and bone metabolism in healthy older adults remain. The age-related decline in glomerular filtration rate (GFR) is central to the belief that high protein intakes are detrimental to kidney function in older adults (Jiang et al. 2012). In opposition, scientific evidence has shown that protein intake increases kidney volume and GFR (Beasley et al. 2014; Skov et al. 1999), without adverse change in renal function (Beasley et al. 2011; Knight et al. 2003). According to the acid-ash hypothesis, it has erroneously been proposed that chronic high protein diets are detrimental to bone health since protein (particularly from animal sources) is an important “acid generating” component of the diet, and structural bone mineral is dissolved to neutralize acids and avoid systemic acidosis (Buclin et al. 2001; Remer and Manz 1994). However, a systematic review and meta-analysis of 35 RCT’s and prospective cohort studies revealed no association and failure to establish causality between protein intake and bone health (assessed by bone calcium retention and bone mineral density) (Fenton et al. 2011). Indeed, several studies have reported a positive relationship between protein intake and peak bone mass in older adults (Hannan et al. 2000; Sahni et al. 2014). In sum, the assertion that higher protein intakes result in negative health outcomes for kidney function and bone metabolism appears largely flawed. To date, the safe upper limit for protein intake in older adults has not been established (Layman et al. 2015); however, based on available evidence, there is no reason to believe that protein recommendations of 1.5 g/kg BM/day will exceed this upper limit for healthy older adults.

## Closing remarks

From both an economic and social perspective, older adults make a positive contribution to today’s society. However, an inevitable consequence of advancing age is the gradual loss of muscle mass and strength, termed sarcopenia. This geriatric condition has known negative impacts on metabolic health, and in later life, the ability to perform acts of daily living. Such outcomes are exacerbated by brief periods of muscle disuse, which unfortunately are experienced with greater frequency in older adults. Currently, interventions to combat sarcopenia are lacking but it is our view that increasing levels of physical activity, resistance exercise in particular, and adequate protein nutrition should be the focus. Specifically, physical activity guidelines for older adults should discourage prolonged periods of muscle disuse and should be refined to include reference to performing some form of resistive-type exercise as being fundamental to preserving muscle mass and function. Future dietary recommendations must also consider ways to increase protein intake whilst taking into account the low appetite levels often experienced by older adults. Thus recommendations should target increasing the utilisation of meal-like quantities of protein for the stimulation of MPS: the key mechanism that regulates maintenance of skeletal muscle mass with advanced age. Future nutritional recommendations for older adults will, we propose, take into consideration the role *other* nutrients (ingested alongside protein) play in sensitising senescent muscle to a protein stimulus. Indeed, emerging evidence suggests that adequate spacing of protein ingestion as well as fortification of protein meals with either leucine or n-3 PUFAs could be a viable means to enhance daily rates of MPS.
